# Supplementary material for: Efficacy and long-term safety of CRISPR/Cas9 genome editing in the SOD1-linked mouse models of ALS
Source: Commun Biol. 2021 Mar 25;4:396. doi: 10.1038/s42003-021-01942-4 (PMC7994668; doi:10.1038/s42003-021-01942-4)
Supplement: Supplementary file 2 — Supplementary Information [file 42003_2021_1942_MOESM2_ESM.pdf]

Supplementary Information

Efficacy and long-term safety of CRISPR/Cas9 genome editing in the *SOD1*-linked mouse models of ALS

Han-Xiang Deng [1*], Hong Zhai [1], Yong Shi [1], Guoxiang Liu [1], Jessica Lowry [1], Bin Liu [1], Éanna B. Ryan [1], Jianhua Yan [1], Yi Yang [1], Nigel Zhang [1], Zhihua Yang [1], Erdong Liu [1], Yongchao Charles Ma [2] and Teepu Siddique [1*].

[1] The Ken and Ruth Davee Department of Neurology, Northwestern University Feinberg School of Medicine, Chicago, IL 60611, USA.

[2] Departments of Pediatrics, Neurology and Physiology, Lurie Children's Hospital of Chicago Research Center, Northwestern University Feinberg School of Medicine, Chicago, IL 60611, USA.

*Correspondence should be addressed to H.-X.D. (h-deng@northwestern.edu) or T.S. (t-siddique@northwestern.edu), The Ken and Ruth Davee Department of Neurology, Northwestern University Feinberg School of Medicine, Tarry Building, Room 13-715, 303 East Chicago Avenue, Chicago, IL 60611, USA.

Supplementary Figures

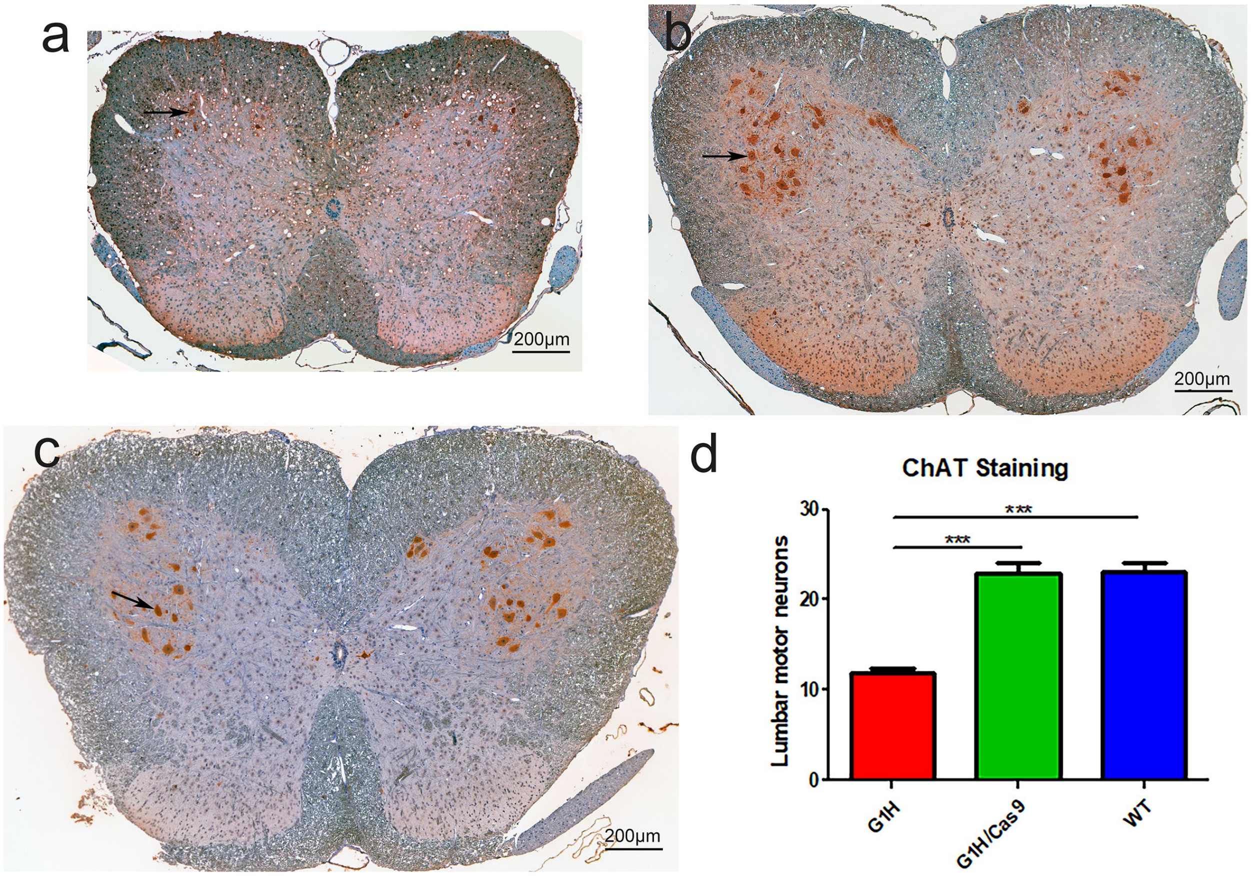

Supplementary Figure 1. Prevention of motor neuron loss by CRISPR/Cas9 editing in G1H/Cas9 mice. Lumbar spinal cord sections were stained with an antibody against choline acetyltransferase (ChAT) by immunohistochemistry. Whole cross-section images are shown for G1H (a), G1H/Cas9 (b) and wild-type (WT, c) mice. Representative ChAT-positive motor neurons are indicated by arrows. Scale bar, 200 μm. Shrinkage of the spinal cord, together with the shrinkage and loss of ChAT-positive neurons in the anterior horns of the G1H mice are shown in panel (a). (d) The number of ChAT-positive neurons in the anterior horns from each of the three genotypes was counted. Three mice were analyzed for each genotype (Supplementary Methods). The mean number of ChAT-positive neurons/section in the anterior horns from G1H (34 sections), G1H/Cas9 (33 sections) and WT (41 sections) is 11.94 ± 2.60, 22.8 ± 6.76 and 23.05 ± 6.38, respectively. The difference of ChAT-positive neuronal number between G1H/Cas9 and WT mice was not statistically significant ($p > 0.05$). *** indicates $p < 0.0001$.

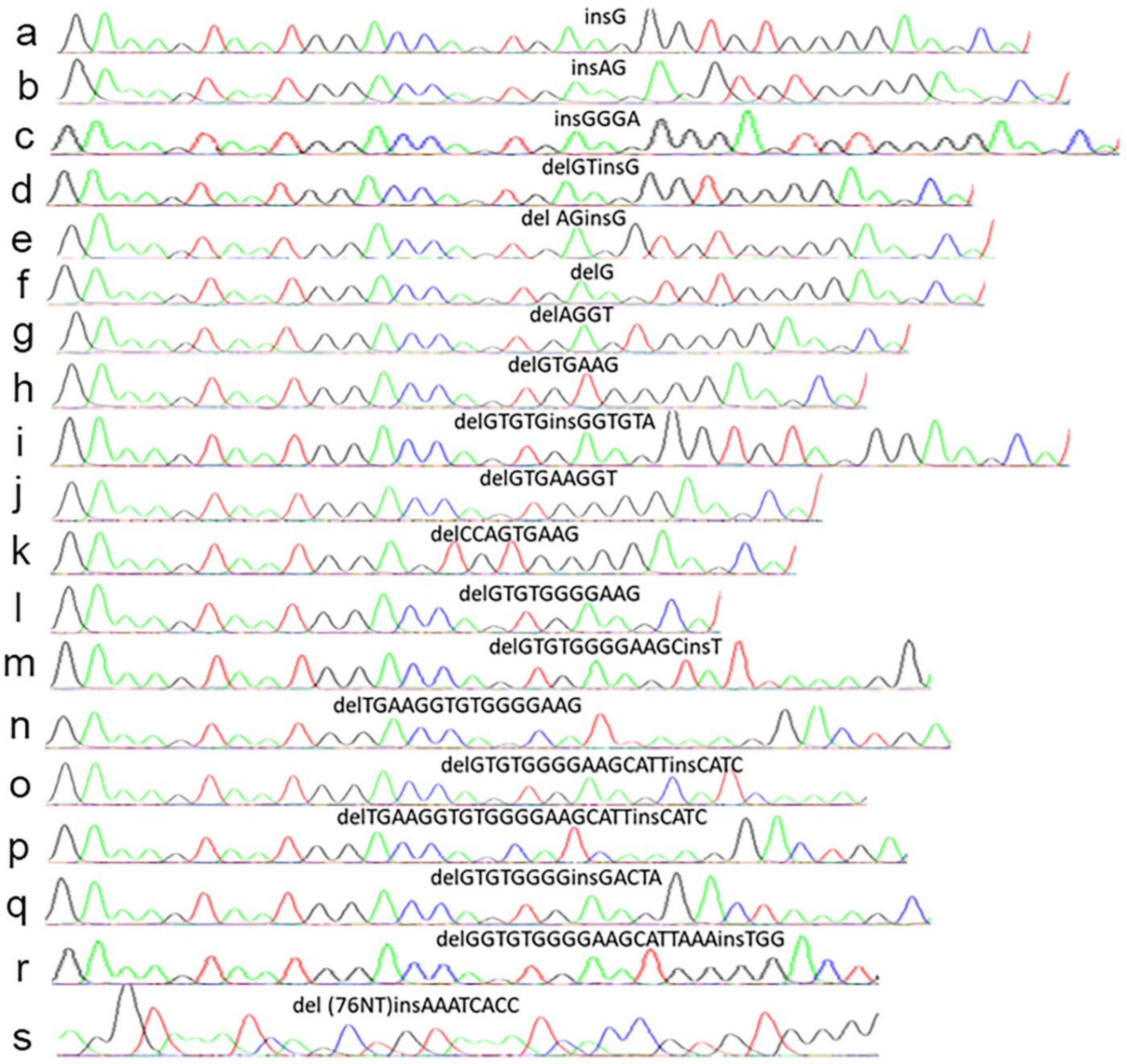

Supplementary Figure 2. Sequencing chromatograms of CRISPR/Cas9 targeted alleles. DNA from a G1H/Cas9 mouse (#8190) was amplified by PCR using primers flanking exon2 (hSOD1-Cas9TP-2F and hSOD1-Cas9TP-2R). The DNA fragments were cloned into a plasmid vector *pBluescript* II SK(-). Individual clones were sequenced. Among 112 individual clones analyzed, 19 different editing events were identified. No wild-type clones were identified. Individual editing events are labeled on the top of the sequencing chromatograms. For the deletion of 76 nucleotides, del (76NT) is labeled.

```
   GAAAGTAATGGACCAGTGAAG----GTGTGGGGAAGCATTAAAGGACT

36 GAAAGTAATGGACCAGTGAA-----GTGTGGGGAAGCATTAAAGGACT delG
 2 GAAAGTAATGGACCAGTGA------GTGTGGGGAAGCATTAAAGGACT delAG
 1 GAAAGTAATGGACCAGTGA-----GGTGTGGGGAAGCATTAAAGGACT delAGinsG
10 GAAAGTAATGGACCAGTGAAG--AGGTGTGGGGAAGCATTAAAGGACT insAG
 4 GAAAGTAATGGACCAG---------GTGTGGGGAAGCATTAAAGGACT delGTGAA
49 GAAAGTAATGGACCA----------GTGTGGGGAAGCATTAAAGGACT delGTGAAG
 3 GAAAGTAATGGAC-----------AGTGTGGGGAAGCATTAAAGGACT delCAGTGAAGinsA
12 GAAAGTAATGGAC---------ACAGTGTGGGGAAGCATTAAAGGACT delCAGTGAAGinsACA
 0 GAAAGTAATGGACCAGTGAAG----GTGTGGGGAAGCATTAAAGGACT WT
```

Supplementary Figure 3. Targeted editing events identified in a G1L/Cas9 mouse (#8306). A total of 117 individual clones derived from the G1L/Cas9 mouse (#8306) were sequenced, and eight different targeted editing events were identified. PAM sequence (TGG) is labeled in green. Red arrowheads indicate the Cas9 cleavage site. The number of clones is shown on the left. Individual mutations are shown on the right. The deleted nucleotides are shown by red dash lines. Red letters represent the inserted nucleotides.

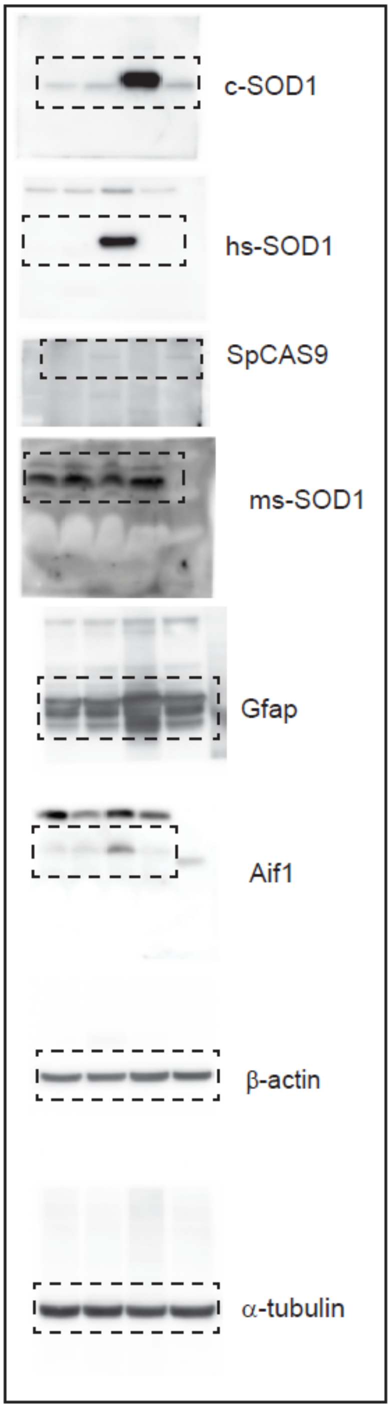

Supplementary Figure 4. Uncropped blot images of Figure 3b. The cropped blot areas shown in Figure 3b are framed. Antibodies are shown on the right.

```
   GAAAGTAATGGACCAGTGAAG--------GTGTGGGGAAGCATTAAAGGACT WT

22 GAAAGTAATGGACCAGTGAAG-------GGTGTGGGGAAGCATTAAAGGACT insG
 9 GAAAGTAATGGACCAGTGAAG------AGGTGTGGGGAAGCATTAAAGGACT insAG
 1 GAAAGTAATGGACCAGTGA---------GGTGTGGGGAAGCATTAAAGGACT delAGinsG
 3 GAAAGTAATGGACCAGTGAA---------GTGTGGGGAAGCATTAAAGGACT delG
16 GAAAGTAATGGACCA--------------GTGTGGGGAAGCATTAAAGGACT delGTGAAG
 1 GAAAGTAATGGACCAGTGAAG-------------------CATTAAAGGACT del11
 1 GAAAGTAATGGACCAG------------------------CATTAAAGGACT del16
 1 GAAAGTAATGGACCAGTGAA------TGG------------------GGACT del19ins3
 3 ---------------------AAATCACCGTGTGGGGAAGCATTAAAGGACT del76ins8
 2 GAAAGTAATGGACCAGTG--------TGG----------------------- del33ins3
 1 --------------AAATCACCGTGTGGA-------GAAGCATTAAAGGACT del83ins15
 1 GAAAGTAATGGACCAGTGA-------CTA----------------------- del133ins3
 0 GAAAGTAATGGACCAGTGAAG--------GTGTGGGGAAGCATTAAAGGACT WT
```

Supplementary Figure 5. Small insertions/deletions in a G1H/Cas9 mouse (#8190) in the clones from long-range PCR products. Among 61 clones with an insert size similar to wild-type clones (type 1, Wt-like), 12 small insertions/deletions were identified, with similar overall frequencies to those in the short-range PCR clones from the same mouse. The PAM sequence (TGG) is labeled green. Red arrowheads indicate the Cas9 cleavage site. The number of clones with the indicated mutation are shown on the left. Individual mutations are shown on the right. The deleted nucleotides are shown by red dash lines. Red letters represent the inserted nucleotides. For the deletions over six nucleotides, the deleted nucleotides are represented by numbers for clarity.

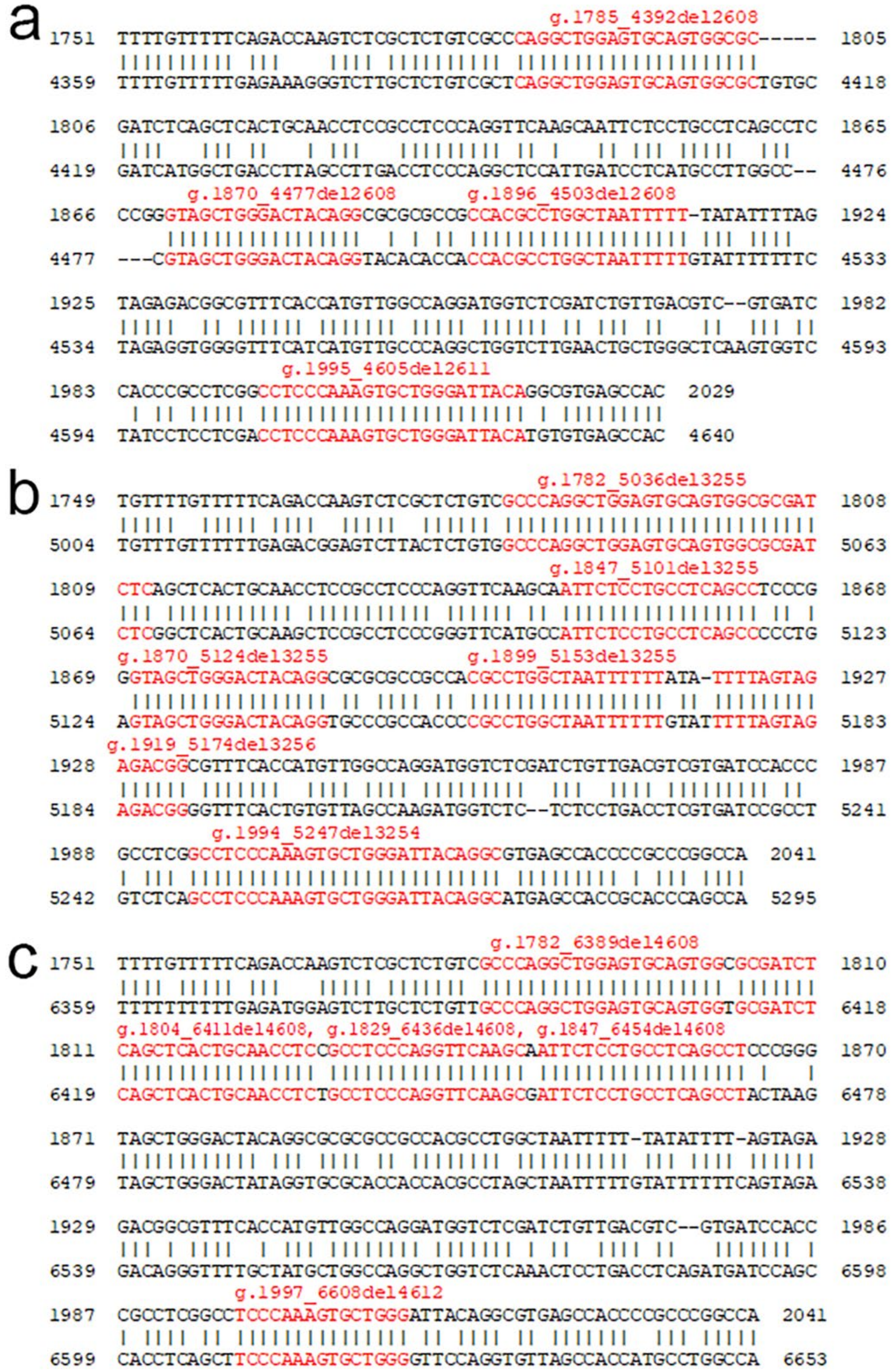

Supplementary Figure 6. Prediction of potential large deletions by PIS flanking the Cas9 cleavage site. The entire *hSOD1* DNA sequence on the 5' arm of the Cas9 cleavage site (4189_4190) was aligned to that on the 3' arm to search for inter-arm PIS. PIS ≥15nt shared by both arms are labeled in red. This alignment revealed one *Alu* element in the 5' arm, which has multiple ASIS in three *Alu* elements in the 3' arm (a-c). Fifteen potential large deletions were predicted, assuming that a PIS ≥15nt in these *Alu* elements might be able to mediate recombination, and to result in large deletions. Description of the deletions is based on the NCBI reference sequence (NC_000021.9) for human *SOD1*.

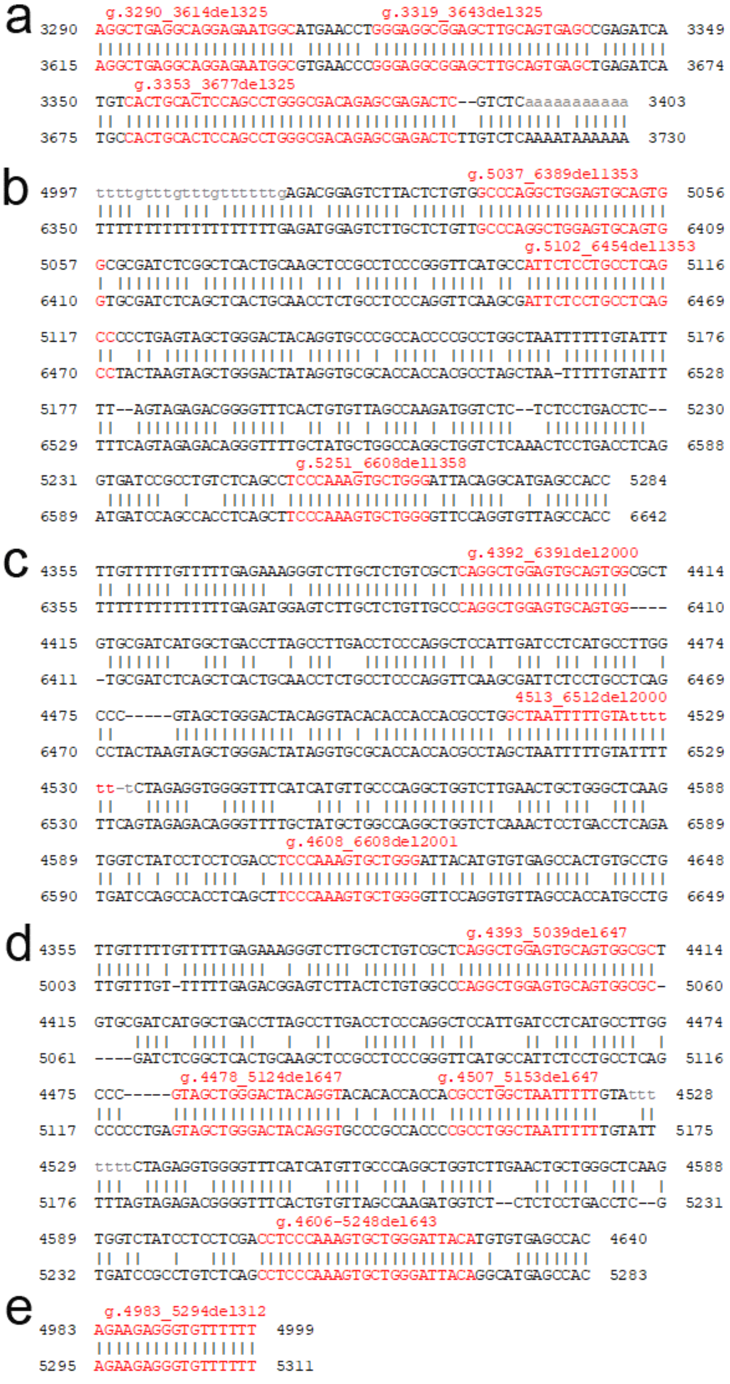

Supplementary Figure 7. Prediction of potential intra-arm deletions by PIS within the arms. The entire *hSOD1* DNA sequence on the 5' arm or 3' arm of the Cas9 cleavage site (4189_4190) was aligned with itself to search for intra-arm PIS. PIS ≥15nt shared within the 5' arm or 3' arm are labeled in red. This alignment revealed two *Alu* elements in each arm. Three (a) and 11 (b-e) potential intra-arm deletions were predicted in the 5' and 3' arms, respectively, assuming that a PIS ≥15nt in these *Alu* elements might be able to mediate intra-arm recombination and to result in deletions. Description of the deletions is based on the NCBI reference sequence (NC_000021.9) for human *SOD1*.
