# Supplementary material for: Efficacy and long-term safety of CRISPR/Cas9 genome editing in the SOD1-linked mouse models of ALS
Source: Commun Biol. 2021 Mar 25;4:396. doi: 10.1038/s42003-021-01942-4 (PMC7994668; doi:10.1038/s42003-021-01942-4)
Supplement: Supplementary file 3 — Reporting Summary [file 42003_2021_1942_MOESM3_ESM.pdf]

nature research

Corresponding author(s): Han-Xiang Deng & Teepu Siddique

Last updated by author(s): Feb 20, 2021

Reporting Summary

Nature Research wishes to improve the reproducibility of the work that we publish. This form provides structure for consistency and transparency in reporting. For further information on Nature Research policies, see our Editorial Policies and the Editorial Policy Checklist.

Statistics

For all statistical analyses, confirm that the following items are present in the figure legend, table legend, main text, or Methods section.

n/a	Confirmed	
☐	☒	The exact sample size (n) for each experimental group/condition, given as a discrete number and unit of measurement
☐	☒	A statement on whether measurements were taken from distinct samples or whether the same sample was measured repeatedly
☐	☒	The statistical test(s) used AND whether they are one- or two-sided<br>*Only common tests should be described solely by name; describe more complex techniques in the Methods section.*
☒	☐	A description of all covariates tested
☒	☐	A description of any assumptions or corrections, such as tests of normality and adjustment for multiple comparisons
☒	☐	A full description of the statistical parameters including central tendency (e.g. means) or other basic estimates (e.g. regression coefficient) AND variation (e.g. standard deviation) or associated estimates of uncertainty (e.g. confidence intervals)
☐	☒	For null hypothesis testing, the test statistic (e.g. F, t, r) with confidence intervals, effect sizes, degrees of freedom and P value noted<br>*Give P values as exact values whenever suitable.*
☒	☐	For Bayesian analysis, information on the choice of priors and Markov chain Monte Carlo settings
☒	☐	For hierarchical and complex designs, identification of the appropriate level for tests and full reporting of outcomes
☒	☐	Estimates of effect sizes (e.g. Cohen's d, Pearson's r), indicating how they were calculated

Our web collection on statistics for biologists contains articles on many of the points above.

Software and code

Policy information about availability of computer code

Data collection	No software was used to collect data.
Data analysis	No software was used to analyze data.

For manuscripts utilizing custom algorithms or software that are central to the research but not yet described in published literature, software must be made available to editors and reviewers. We strongly encourage code deposition in a community repository (e.g. GitHub). See the Nature Research guidelines for submitting code & software for further information.

Data

Policy information about availability of data

All manuscripts must include a data availability statement. This statement should provide the following information, where applicable:

- Accession codes, unique identifiers, or web links for publicly available datasets
- A list of figures that have associated raw data
- A description of any restrictions on data availability

We did not use publicly available datasets to generate data in this study. There are no restrictions to use the data in this paper.

Field-specific reporting

Please select the one below that is the best fit for your research. If you are not sure, read the appropriate sections before making your selection.

☒ Life sciences ☐ Behavioural & social sciences ☐ Ecological, evolutionary & environmental sciences

For a reference copy of the document with all sections, see nature.com/documents/nr-reporting-summary-flat.pdf

Life sciences study design

All studies must disclose on these points even when the disclosure is negative.

Sample size	For survival analysis, fourteen G1H and 15 G1H/Cas9 mice were included. For counting motor neurons in the spinal cord, three mice for each genotype, including wild-type, G1H and G1H/Cas9, were included.
Data exclusions	No data were excluded from the analysis.
Replication	No replication was performed.
Randomization	No randomization was involved in this study
Blinding	Researcher were blinded during experiments. Different researchers performed either mouse genotype or phenotype characterization independently.

Reporting for specific materials, systems and methods

We require information from authors about some types of materials, experimental systems and methods used in many studies. Here, indicate whether each material, system or method listed is relevant to your study. If you are not sure if a list item applies to your research, read the appropriate section before selecting a response.

Materials & experimental systems

n/a	Involved in the study	
☐	☒	Antibodies
☒	☐	Eukaryotic cell lines
☒	☐	Palaeontology and archaeology
☐	☒	Animals and other organisms
☒	☐	Human research participants
☒	☐	Clinical data
☒	☐	Dual use research of concern

Methods

n/a	Involved in the study	
☒	☐	ChIP-seq
☒	☐	Flow cytometry
☒	☐	MRI-based neuroimaging

Antibodies

Antibodies used	Primary antibodies used in this study included SpCas9 (ab191468, Abcam, Cambridge, UK), GFAP (G4546, Sigma-Aldrich, Inc., St. Louis, MO) , IBA1/AIF1 (#MABN92, Millipore Sigma-Aldrich, Inc., St. Louis, MO), β-actin (A5060, Sigma-Aldrich, Inc., St. Louis, MO), α-tubulin (#66031-1-Ig, Proteintech Group, Chicago, IL), ubiquitin (#10201-2-AP, Proteintech Group, Chicago, IL) and CHAT (#AB144P, Millipore Sigma-Aldrich, Inc., St. Louis, MO). The fluorescent secondary antibodies included Alexa Fluro 488 goat anti-mouse IgG (A11029), Alexa Fluro 488 goat anti-rabbit IgG (A11034), Alexa Fluro 555 goat anti-mouse IgG (A21424), Alexa Fluro 555 goat anti-rabbit IgG (A21429) from the Life Technologies/Invitrogen, Grand Island, NY. In addition, three home-made antibodies to human and mouse SOD1 were used, including hs-SOD1, ms-Sod1 and c-SOD1.
Validation	The profile and validation of each commercially available antibody is publicly available from the manufactures' websites. The home-made antibody (hs-SOD1 and c-SOD1) were validated in our previous study (Deng et al., PNAS 2006).

Animals and other organisms

Policy information about studies involving animals; ARRIVE guidelines recommended for reporting animal research

Laboratory animals	Laboratory mice were included in this study (strain: C57BL/6J, both male and females were included, ranging from 1 to 33 month).
Wild animals	No wild-type animals were used in this study.
Field-collected samples	The study did not involve samples collected from the field.

Ethics oversight	The animals and animal use protocols have been approved by the Institutional Animal Care and Use Committee (IACUC) of Northwestern University.

Note that full information on the approval of the study protocol must also be provided in the manuscript.
